# High-concentration methane and ethane QEPAS detection employing partial least squares regression to filter out energy relaxation dependence on gas matrix composition

**DOI:** 10.1016/j.pacs.2022.100349

**Published:** 2022-03-21

**Authors:** Giansergio Menduni, Andrea Zifarelli, Angelo Sampaolo, Pietro Patimisco, Marilena Giglio, Nicola Amoroso, Hongpeng Wu, Lei Dong, Roberto Bellotti, Vincenzo Spagnolo

**Affiliations:** a PolySense Lab, Dipartimento Interateneo di Fisica M. Merlin, Università degli Studi di Bari Aldo Moro e Politecnico di Bari, Via G. Amendola 173, Bari, 70125, Italy; b PolySense Innovations srl, Via Amendola 173, Bari 70126, Italy; c Dipartimento di Farmacia—Scienze del Farmaco, Università degli Studi di Bari Aldo Moro, Via A. Orabona 4, Bari, 70125, Italy; d State Key Laboratory of Quantum Optics and Quantum Optics Devices, Institute of Laser Spectroscopy & Collaborative Innovation Center of Extreme Optics, Shanxi University, Taiyuan 030006, China; e Dipartimento Interateneo di Fisica M. Merlin, Università degli Studi di Bari Aldo Moro, Via G. Amendola 173, Bari, 70125, Italy

**Keywords:** *Full investigation of CH4, C2H6 signal dependence on the relaxation dynamics within, Gas matrix containing CH4, C2H6, C3H8, With concentrations exceeding the part-perthousand, Concentration range up to several percent.*PLSR employed as a reliable stat, *Sensing system architecture optimized for on-field, Measurements and the data acquisition automatized. The whole system consists in a, Shoebox sized QEPAS sensor, Ready to be deployed for in situ operations, *Potentiality of CH4, C2H6 QEPAS detection over a dynamic range extending from ppb, Scale up to percent scale in natural gas-like mixtures

## Abstract

A quartz enhanced photoacoustic spectroscopy (QEPAS) sensor capable to detect high concentrations of methane (C1) and ethane (C2) is here reported. The hydrocarbons fingerprint region around 3 µm was exploited using an interband cascade laser (ICL). A standard quartz tuning fork (QTF) coupled with two resonator tubes was used to detect the photoacoustic signal generated by the target molecules. Employing dedicated electronic boards to both control the laser source and collect the QTF signal, a shoe-box sized QEPAS sensor was realized. All the generated mixtures were downstream humidified to remove the influence of water vapor on the target gases. Several natural gas-like samples were generated and subsequently diluted 1:10 in N_2_. In the concentration ranges under investigation (1%−10% for C1 and 0.1%−1% for C2), both linear and nonlinear responses of the sensor were measured and signal variations due to matrix effects were observed. Partial least squares regression (PLSR) was employed as a multivariate statistical tool to accurately determine the concentrations of C1 and C2 in the mixtures, compensating the matrix relaxation effects. The achieved results extend the range of C1 and C2 concentrations detectable by QEPAS technique up to the percent scale.

## 1 Introduction

The detection and analysis of hydrocarbon gas samples are crucial tasks for key-field applications such as petroleum exploration [1], planetary geology [2], and monitoring of both terrestrial and marine environments [3]. Natural gas flowing towards Earth’s surface can be used as a probe for exploration below and above the ground, representing a powerful tool for geological and petroleum explorations [4]. These gaseous emissions are primarily formed by methane (C1 > 70%), and can also include ethane (C2), propane (C3), butane (C4) and pentane (C5), which are the results of microbial or thermal conversion of organic matter [5], [6]. Hydrocarbons composing natural gas can be present in a widely variable range of concentrations depending on the origin of the reservoir and on the subsurface geological composition [7], [8]. The real-time collection and analysis of subterranean gas samples would provide a precise mapping of the natural gas reservoirs beneath the surface, thus allowing a geo-steering approach for the borehole placement [9]. The geochemical analysis of the natural gas extracted during drilling operations would then improve the forecasting efficiency in petroleum exploration providing a two-fold advantage: a sensible reduction of the perforation costs and a strong reduction of the impact on environment.

The analysis of natural gas samples is mainly performed exploiting the well-established gas chromatography analysis empowered by flame ionization detectors. Up to now, this analytical method represents the benchmark technique for high concentrations hydrocarbon detection, due to the wide range of operating concentrations and to the high selectivity in multi-gas analysis [10], [11]. Optical detection techniques have emerged as a suitable solution for in situ detection of hydrocarbons in the gas phase. High resolution spectrometers exploiting cavity ring-down spectroscopy (CRDS) [12], off-axis integrated cavity output spectroscopy (OA-ICOS) [13], tunable diode laser absorption spectroscopy (TDLAS) [14] and photoacoustic spectroscopy (PAS) [15], have been used to detect light hydrocarbons, C1 and C2, in the near and mid IR spectral range. The fundamental absorption bands related to C-H bond stretching are located at λ = 3–4 µm, while first overtones can be found at λ = 1–2 µm. Both spectral ranges are easily accessible with highly powerful semiconductor lasers. For this reason, IR optical sensors have been widely employed in ground detection and remote sensing applications to detect hydrocarbon traces, with concentrations in the range of part-per-million (ppm) and part-per-billion (ppb), or even below [16], [17]. Advances in optical sensing techniques focused on the detection of trace gas concentrations, while the high concentration range was barely explored. Up to now, a solid detection approach for high concentration C1 detection had been discussed in [18], where an optical spectrometer based on OA-ICOS demonstrated to detect the target gas up to percent scale. Nevertheless, the possibility to perform multi-gas detection on hydrocarbons sample employing optical sensors is still precluded.

Quartz enhanced photoacoustic spectroscopy (QEPAS) technique is a development of traditional PAS and employs a quartz tuning fork (QTF) as sharply resonant photoacoustic transducer. Laser light resonant with a radiative transition of the target molecule is focused between the QTF prongs, and it is modulated at the QTF resonance frequency or one of its subharmonics. Then, the acoustic waves generated by the modulated relaxation of the target gas are detected and converted into an electric signal, exploiting the piezoelectric properties of QTFs [19]. A pair of resonator tubes is coupled with the QTF to amplify the acoustic waves generated by gas relaxation, composing the so called QEPAS spectrophone. Differently from the above-mentioned detection techniques, QEPAS sensing does not need any optical detector and the sound wave detection by the QTF is wavelength independent. This makes the QEPAS technique suitable to be employed with laser sources characterized by a wide emission range, by exploiting custom QTFs [20], [21]. QEPAS sensors have already demonstrated to be a solid solution to detect hydrocarbon traces in nitrogen (N_2_) matrices for environmental monitoring applications [22], [23]. The high level of compactness and robustness makes QEPAS sensors promising candidates also for development of downhole devices, devoted to the analysis of high concentration hydrocarbons resulting from drilling operations.

Recently, new QEPAS sensors for the detection of C1, C2 and C3 in trace concentrations were proposed, exploiting an interband cascade laser (ICL) emitting at a central wavelength of 3345 nm and a standard 32 kHz QTF, coupled with a pair of optimized resonator tubes [24], [25]. C1 and C2 detection at concentrations ranging from few ppb to 1000 ppm was demonstrated. These two analytes are the main components of natural gas samples, and their concentrations represent the first indicator for the characterization of natural gas reservoirs [4], [7]. In general, only the low concentration region (up to 1000 ppm) has been fully investigated, while there is still lack of a detailed investigation of the response of optical sensors at high concentrations. For on-field explorations, the possibility to employ a single sensor system capable of analyzing gas samples with hydrocarbon concentration varying from ppb to several percent would represent a great value. Dealing with analytes at high concentrations, two different nonlinear behaviors can be observed, related to: i) power losses due to intense optical absorption and ii) photoacoustic wave generation and detection. In the first case, the possibility to rely on the linear approximation of the Lambert-Beer law to describe the optical absorption in the whole spectrum under investigation may be precluded. Thus, the QEPAS signal must be necessarily modelled including the optical power exponential decrease prescribed by the Lambert-Beer law. Therefore, based on these two physical quantities, the response of optical sensors could be linear or nonlinear with respect to the analyte concentration. In the second case, the gas matrix composition plays a key role. In fact, the photoacoustic generation depends on the target molecules de-excitation, occurring in the mid-IR via vibro-translational (V-T) and vibro-vibrational (V-V) energy relaxations through the collisional partners within the mixture, based on the energetic levels of the components within the sample [26]. The parameter representing the efficiency in photoacoustic generation is the radiation-to-sound conversion efficiency *ε(P)*, which is a function of the operating pressure *P* [27]. In addition, strong variations in the gas matrix composition result in variation of the fluid dynamics parameters, thus influencing the resonance characteristics of the QTF, i.e., resonance frequency and Q-factor [28]. Overall, the photoacoustic signal can be modelled as:

(1) S ~ α∙ P_0_ ∙ e^-α ∙ L^ ∙Q ∙ ε

where *α* is the absorption coefficient and *L* is the optical path, Q is the QTF quality factor and P_0_ is the laser power [29].

This last effect on the photoacoustic signal is expressed in terms of radiation-to-sound conversion efficiency, which represents the capability of the excited molecules to convert the absorbed energy in sound [30]. Therefore, if the variability of the gas samples’ composition is large, these effects may strongly affect the sensor response.

When traces of the target analytes are detected within a N_2_ gas matrix, the most common approach to retrieve gas concentrations is to employ linear regression based on a peak value calibration of the QEPAS response. If no correlation among the target gases is observed, this tool can be extended to multi-gas detection by performing a multi-linear regression (MLR) based on the ordinary least squares (OLS) method [27]. When analytes within complex gas matrices are targeted, it is crucial to level off the influence of the gas matrix fluctuations on the photoacoustic signals. In order to achieve this, different approaches can be adopted, such as pursuing an analytical modelling of the matrix effects [31] or developing complex experimental configurations and detection schemes to characterize and compensate the gas matrix variations [32]. The first approach requires a detailed investigation on all the parameters influencing the photoacoustic signal generation, from the thermodynamical parameters to the energy relaxation dynamics in fluctuating backgrounds. The second approach employs two different sensitive elements: a bare standard QTF mainly dedicated to detect the gas matrix macroscopic variations, and a standard spectrophone to perform low concentration measurements. Then, a sophisticated analysis, based on an algorithm of sequential signal comparisons, helps to interpret and take into account all the nonlinearities arising from i) the energy relaxation dynamics and from ii) the change of the speed of sound with consequent degradation of the acoustic resonator tubes amplification factor. More in details, the optimal geometrical parameters, and the length in particular of the resonator tubes, mainly depend on the sound wavelength [33]. Therefore, a change of the sound velocity due to a matrix variation will influence the response of the QEPAS spectrophone in terms of Q-factor and resonance frequency, resulting in a variation of the signal amplitude. These ineliminable drawbacks, peculiar of the photoacoustic technique, may discourage its exploitation in facing the challenge of the wide range detection, which is a requirement shared by the almost totality of the modern sensing technologies. This would mean giving up on a compact, robust, and highly deployable architecture like that of QEPAS systems.

Nevertheless, an alternative calibration approach relying on statistics rather than affording a full characterization of each physical phenomenon affecting the photoacoustic detection, can definitely help in overcoming the gas matrix issue. In fact, if there is correlation among the detected molecules the MLR approach is no longer reliable, and a different kind of multivariate analysis (MVA) is required. A suitable choice to retrieve analytes concentration in correlated gas samples is the partial least squares regression (PLSR). PLSR is a MVA approach developed as a generalization of MLR, able to analyze data with correlated and noisy variables [34], which was already validated as a solid tool for QEPAS sensing to characterize gas species with highly overlapping absorption spectra [35].

In this work, we use the interesting study case of C1, C2 detection range extension up the percent scale as a bench test for validating the suitability of the PLSR analysis at filtering out the energy relaxation dependence on gas matrix composition, representing a novel approach for the analysis of complex gas samples. A detection scheme employing an ICL and a standard QEPAS spectrophone was selected. The analysis was performed by calibrating and testing the regression model on custom gas samples, realized mixing certified concentrations of the target gases, with C1 concentration ranging from 1% to 10%, and C2 concentration from 0.1% to 1%, in nitrogen. The regression tool was then blind tested using a certified natural gas mixture, in a 1:10 dilution. The influence of heavier hydrocarbons on the signal generation was evaluated including gas mixtures containing C3 at different concentrations in the calibration step. The achieved results demonstrated the ability of the employed sensing scheme to remove the influence of matrix relaxation effects and return the C1 and C2 concentrations within the analyzed samples.

## 2 Experimental setup

**Fig. 1:**
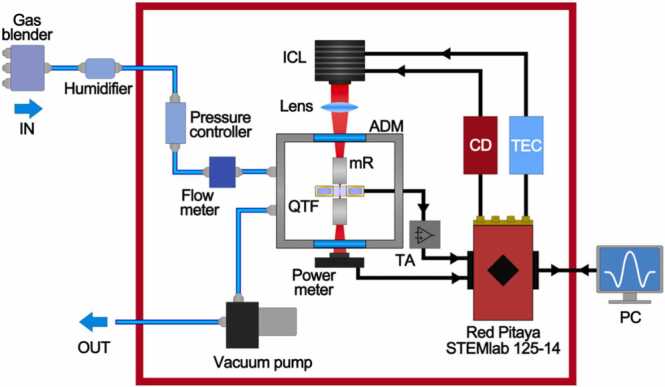
Schematic of the experimental apparatus: ICL - Interband Cascade Laser, ADM - Acoustic Detection Module, mR –resonator tubes, QTF - Quartz Tuning Fork, TA – Transimpedance Amplifier, CD – Current Driver, TEC – ThermoElectric Cooler, PC – Personal Computer.

The experimental setup represented in Fig. 1 was employed to acquire the QEPAS spectra of mixtures containing high concentrations of C1 and C2.

In this work, we employed a Nanoplus ICL, with central emission wavelength of 3345 nm (2989 cm^−1^) and operating in the 20–30 °C temperature range. A maximum emitted power of ~ 8 mW was measured when working at the selected temperature of T = 27.5 °C. The laser source was driven using compact, low-consumptions, electronic boards instead of traditional laser drivers. A Red Pitaya STEMlab 125–14 board was employed to control a custom Thorlabs ICL current driver and a MTD1020T thermoelectric cooler. The former provided the injection current to the laser source, while the latter set and monitored the operating temperature. A custom LabVIEW-based software was used to control the electronic boards. A standard spectrophone composed of a standard QTF and a pair of resonator tubes was placed inside an acoustic detection module (ADM). The fundamental mode resonance frequency and quality factor at atmospheric pressure in pure N_2_ were measured as f_0_ = 32741.5 Hz and 2000, respectively.

All the measurements were collected using *2 f-*wavelength modulation (WM) technique, modulating the laser current with a frequency of f_0_/2, and acquiring the f_0_-oscillating component of the spectrophone signal output. A current sweep was superimposed to the sinusoidal modulation to perform spectral scans in the laser dynamic range. Both the injection current modulation and the QTF signal demodulation were performed using a custom LabVIEW-based software and a lock-in subroutine.

The measurements were carried out at a working pressure of 760 Torr and at a flow rate of 20 sccm. These conditions were controlled and fixed using an Alicat EPC-15PSIA-P01 pressure controller, an Axetris MFM 2220-BA-U0 flow meter and a Thomas 1420BLDC diaphragm pump. Mixtures of hydrocarbons were generated starting from certified concentration gas cylinders, presenting a relative 4% uncertainty, connected to an MCQ Instruments Gas Blender GB-103. A Nafion humidifier was placed downstream the gas blender to stabilize the absolute humidity in the mixtures at a concentration of 2%.

Aiming to develop a compact sensor for in situ operation, the employed configuration moved from a laboratory-based benchtop scheme towards a portable design. Therefore, the whole developed QEPAS sensor, except for the gas mixer and the humidifier, fitted within a shoe-box size aluminum crate (schematically shown as a red square in Fig. 1).

## 3 Methane and ethane calibration

**Fig. 2:**
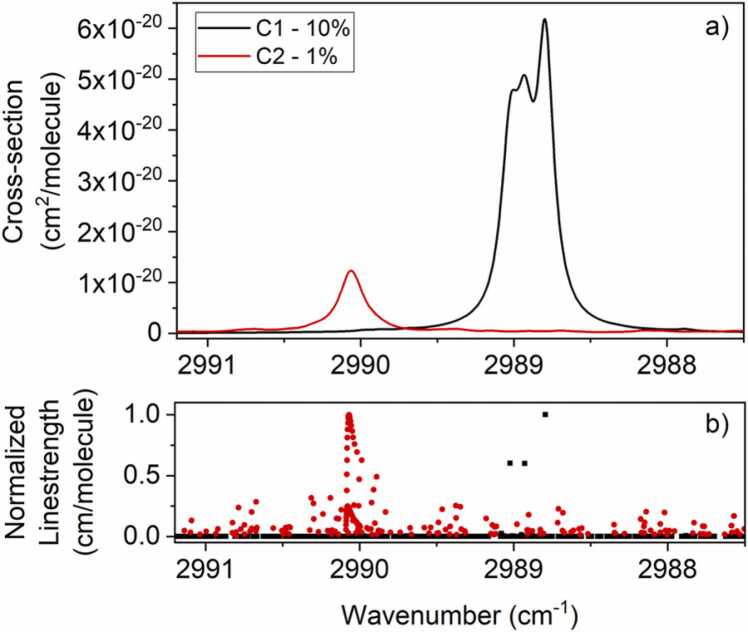
a) absorption cross-sections at a pressure of 760 Torr for two gas samples containing 10% of C1:N_2_ (black curve) and 1% of C_2_:N_2_ (red curve), respectively, in the wavenumber range 2987.5–2991.2 cm^−1^ simulated though HITRAN database [37] b) C1 (black dots) and C2 (red dots) corresponding normalized linestrengths. (For interpretation of the references to colour in this figure legend, the reader is referred to the web version of this article.)

In this section, the absorption spectra of C1 and C2 are reported as well as the corresponding QEPAS signals and calibration curves. All measurements were performed at atmospheric pressure, which provides a twofold advantage: i) the equilibrium between internal and external pressure avoids even the minimum leak and allows continuous flow measurements as well as static measurements, if needed; ii) radiation-to-sound conversion efficiency *ε(P)* improves as the total pressure increases [36]. HITRAN database was employed to simulate two gas samples containing 10% of C1:N_2_ and 1% of C2:N_2_, respectively, in the laser dynamic range at atmospheric pressure, and the results are shown in Fig. 2.

The main result of working at atmospheric pressure is the merging of several optical transitions into broader absorption structures, as it can be easily noticed for the three C1 absorption lines falling at 2988.9 cm^−1^, 2989.0 cm^−1^ and 2988.8 cm^−1^ (black dots in Fig. 2**b**). A similar situation arises for C2 spectrum, where the absorption cross-section (red curve in Fig. 2**a**) shows an absorption feature resulting from the merging of several lines at ~ 2990 cm^−1^, as well as several structures with lower intensity distributed all over the laser dynamic range (red dots in Fig. 2**b**).

**Fig. 3:**
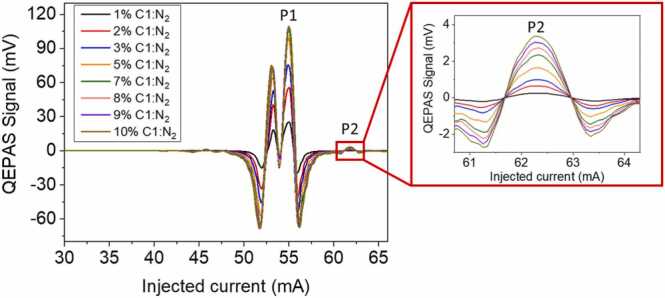
Measured QEPAS signals for different C1:N_2_ concentrations. The trends of the peaks P1 and P2, falling at 55 mA and at 61.8 mA, respectively, were selected as C1 calibration curves. In the inset, a zoom of the P2 peak is shown.

Methane is the first analyte under investigation and its 2 f-QEPAS spectral scans are shown in Fig. 3**,** as a function of the laser injection current for different concentrations, ranging from 1% to 10% of C1 in N_2_. Hereafter, all the generated gas samples are downstream humidified with a fixed water vapor concentration.

**Fig. 4:**
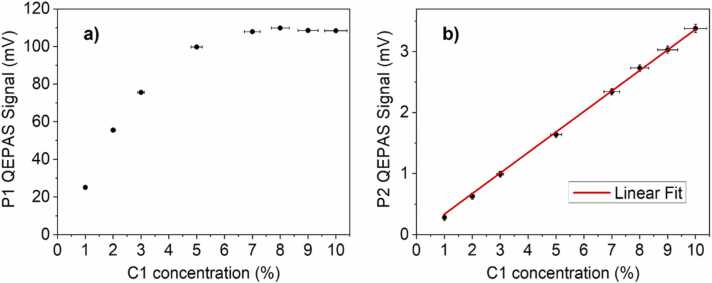
Calibration curves of the selected P1 (a) and P2 (b) peaks in the concentration range 1–10% in C1:N_2_ mixtures. The error bars are calculated starting from a measured relative error on the QEPAS signal of 2% and a relative error on C1 concentration of 4% estimated from the extended uncertainty of the certified gas cylinders employed.

As a result of the spectroscopic scenario shown in Fig. 2a, within the laser dynamic range (Fig. 3**)** C1 QEPAS signal is clearly recognizable in two current ranges, i.e., 47.5–60 mA and 60–64 mA. In the first current range, two methane peaks are well resolved and have comparable signal values. The detection phase of P1 peak, falling at 55 mA, was used as a reference for acquiring the QEPAS spectra of all the hydrocarbon mixtures analyzed in the following. This feature corresponds to the absorption line located at 2988.8 cm^−1^, while, the peak falling at 53 mA corresponds to the merged absorption features at ~ 2989.0 cm^−1^ as shown in Fig. 2. The wavelength modulation depth employed for 2 f-QEPAS detection was selected to maximize and resolve P1 from the neighbor peak. The C1 peak P2, falling at 61.8 mA, is detectable in the second current range (inset of Fig. 3) and exhibits a signal intensity more than 10 times lower than P1. This feature corresponds to a weak absorption line located at 2987.9 cm^−1^ [37]. The calibration curves of both P1 and P2 methane peaks are shown in Fig. 4, in which each data point is reported with signal and concentration error bar.

The nonlinearly increasing signal for C1 concentration, ranging from 1% up to 7%, and the plateau reached at ~110 mV are perfectly consistent with Eq. 1. In fact, P1 peak calibration curve (Fig. 4**a**) clearly exhibits the photoacoustic signal dependence both on the absorption coefficient and on the residual laser power surviving the direct absorption within the ADM and available for photoacoustic generation. Conversely, P2 QEPAS signal (Fig. 4**b**) linearly depends on C1 concentration in the range under investigation, and the best linear fit returned a slope of 0.35 mV/% and a R^2^ of 0.9996.

**Fig. 5:**
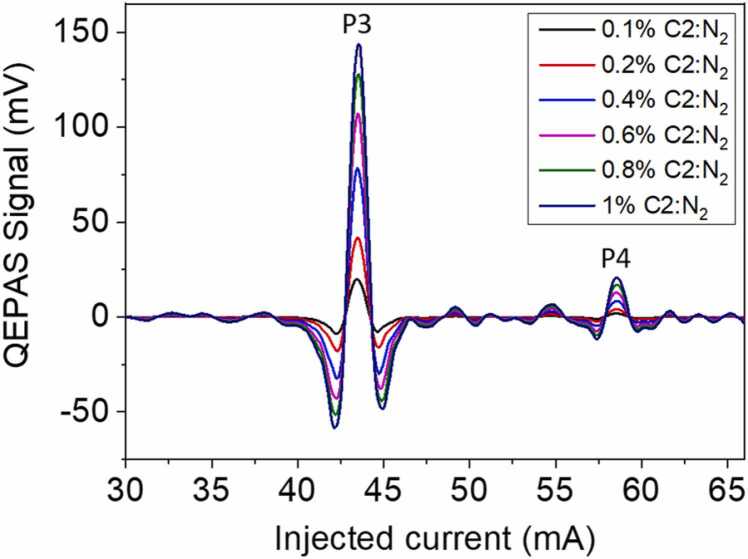
Measured QEPAS spectra for different C2:N_2_ concentrations.

The second analyte under investigation is ethane and the QEPAS signal is analyzed within the 0.1%− 1% concentration range, as reported in Fig. 5.

**Fig. 6:**
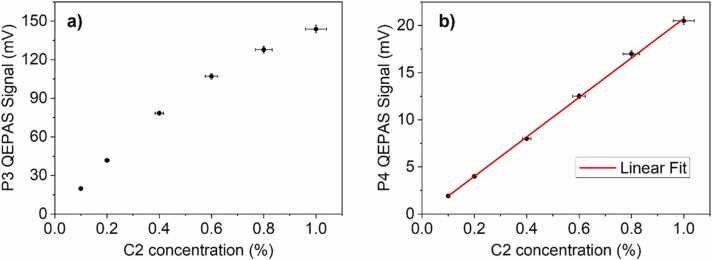
Calibration curves of the selected P3 (a) and P4 (b) C2 peaks in the concentration range 0.1–1% in C2:N_2_ mixtures. The error bars are calculated starting from a measured relative error on QEPAS signal of 2% and a relative concentration error of 4%.

The C2 QEPAS spectra show two clearly distinguishable peaks, labelled as P3 and P4, and several weaker absorption features already observed in previous experiments [24]. The P3 peak falls at 43.5 mA, corresponding to the merged ethane absorption lines located nearby 2990 cm^−1^, and does not directly interfere with P1, P2 methane features. Conversely, the P4 peak falling at 58.5 mA could overlap with P1 peak of C1 falling at 55 mA in a mixture containing both CH_4_ and C_2_H_6_ molecules. The calibration curves for both P3 and P4 are represented in Fig. 6.

As in the case of the highest C1 peak, P3 peak calibration curve (Fig. 6**a**) exhibits a nonlinear trend but, differently from P1, no saturation occurs and the QEPAS signal is a monotonic function of the concentration in the 0.1%− 1% range. The P4 peak (Fig. 6**b**) increases linearly in the concentration range under investigation and the best linear fit function has a slope of 20.92 mV/% and a R^2^ of 0.9993.

## 4 Propane spectral interference

**Fig. 7:**
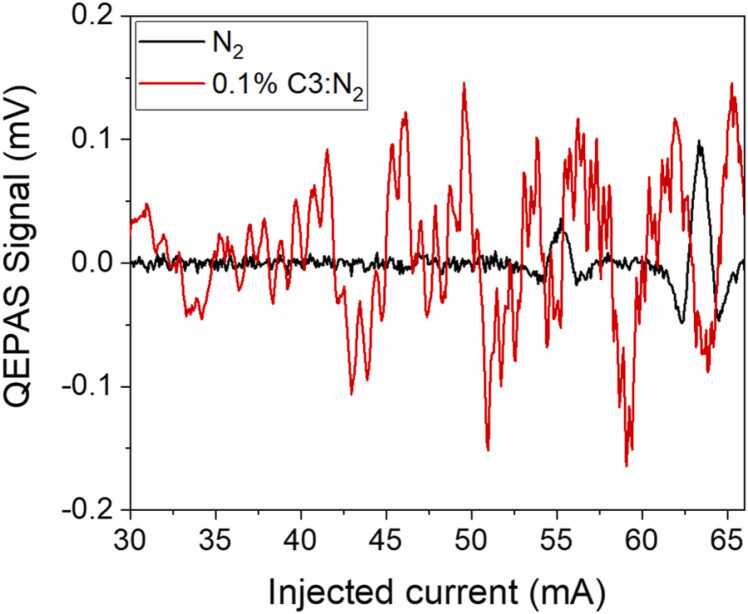
Comparison between the measured QEPAS signal of pure nitrogen (black curve) and a 0.1% C3:N2 mixture (red curve). Two water vapor absorption lines, falling at 55.2 mA and at 63.3 mA, are identified in the laser dynamic range. (For interpretation of the references to colour in this figure legend, the reader is referred to the web version of this article.)

In natural gas-like mixtures, the presence of hydrocarbon molecules more structured than C1 or C2, such as propane (C3) and butane (C4), is expected but in lower concentration [38]. For this investigation, C3 was chosen as a representative analyte for all the more complex alkanes and non-hydrocarbon components that could be found in natural gas and potentially affect the photoacoustic detection of C1 and C2. Trace detection of propane was already demonstrated and the interference among the absorption spectra of C1, C2 and C3 was investigated [25]. In the spectral region from 3.3 to 3.5 µm the absorption spectrum of C3 appears as broad, non-flat, absorption band composed of several absorption features merged together with a sharp absorption peak located at 3369.76 nm, according to the PNNL spectral database [39]. The intensity of the C3 merged features is significantly lower compared to those of C1 and C2 in the same spectral range. In Fig. 7, a comparison between the QEPAS signals of a 0.1% C3 in nitrogen mixture and a pure nitrogen sample is shown*.* Both measurements were collected optimizing the gain of the transimpedance amplifier, thus allowing the detection of the signals in the µV range with the setup reported in Fig. 1. For ease of comprehension, the acquired spectra were rescaled to be compared with the C1 and C2 measurements collected in low-gain configuration.

The combination of the low absorption intensities and the broadened lineshape returns an irregular, low signal 2 f-QEPAS spectrum, still recognizable with respect to the N_2_ background signal and similar to the one observed in previous investigations [25]. The QEPAS signal for a gas sample of wet nitrogen (black curve in Fig. 7) allows the measurement of the noise level, considered as the standard deviation of the acquired data, as low as 3.7 μV in the 30–50 mA range. The red curve in Fig. 7 represents the measured QEPAS signal of a 0.1% C3 in N_2_ mixture. Although propane signal is well recognizable with respect to ground noise with a standard deviation of 44.4 μV, the spectral interference with respect to C1 (Fig. 3) and C2 (Fig. 5) signals is negligible. Same considerations apply for the signal due to the two water absorptions at 55.2 mA and 63.3 mA.

## 5 Ethane and propane effects on methane QEPAS signal

**Fig. 8:**
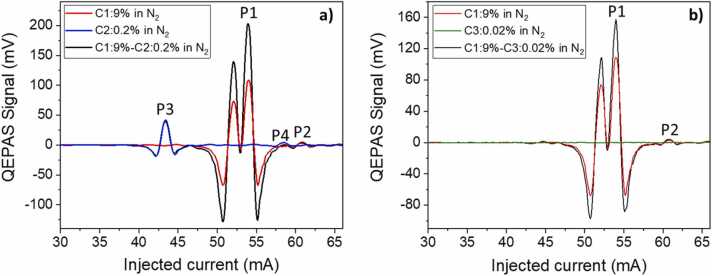
a) Comparison between the QEPAS signals of both 9% of C1 in N2 (red curve) and 0.2% of C2 in N2 (blue curve) with the signal of a mixture of 9% of C1 and 0.2% of C2 in N2 (black curve). b) Comparison between the QEPAS signals of both 9% of C1 in N2 (red curve) and 0.02% of C3 in N2 (green curve) with the signal of a mixture of 9% of C1 and 0.02% of C3 in N2 (black curve). (For interpretation of the references to colour in this figure legend, the reader is referred to the web version of this article.)

The QEPAS signal of a target molecule depends on the energy relaxation pathways with the different collisional partners, i.e., on the mixture composition. This effect is strongly evident for slow-relaxing molecules, such as methane. In fact, C1 QEPAS signal proved to be dependent on the water vapor concentration in the 3 µm wavelength range [40], therefore the H_2_O concentration was fixed employing a Nafion humidifier in the experimental setup (Fig. 1). In this section, the effect of C2 and C3 on the C1 QEPAS signal was investigated. QEPAS signals of C1-C2 and C1-C3 mixtures were analyzed to study C1 dependence on the other hydrocarbons under investigation. Two representative mixtures of C1-C2 and C1-C3 are shown in Fig. 8, together with single-gas calibration QEPAS spectra.

Firstly, the effect of C2 concentration on C1 QEPAS signal was investigated, comparing the spectra of a two-component mixture C1 9%:C2 0.2%:N_2_, with respect to the single-component mixtures C1 9%:N_2_ and C2 0.2%:N_2_. In the two-component spectrum, the absorption features belonging to each molecule are clearly recognizable, as expected from the HITRAN simulation shown in Fig. 2. In Fig. 8**a**, even if C1 concentration does not change between the red curve (C1 9%:N_2_) and the black curve (C1 9%:C2 0.2%:N_2_), P1 peak signal increases from 108.50 mV to 203.49 mV when C2 is added to the N_2_ gas matrix, while C2 background absorption signal is lower than 2 mV in the 47.5–60 mA current range for the C2 concentration range under investigation (Fig. 5). Therefore, the increase of P1 value can be addressed to the different relaxation dynamics of C1 within a C1-C2 mixture. Moreover, since the C1 QEPAS signal is lower than 2 mV in the 40–45 mA current range, P3 peak value can be assumed to depend only on C2 concentration. Indeed, P3 peak value does not change from C2 0.2%:N_2_ to C1 9%:C2 0.2%:N_2_ mixture, thus the presence of C1 does not influence the relaxation dynamics of C2 within the gas matrix. It is possible to identify P2 and P4 peaks in the C1 9%:C2 0.2%:N_2_ mixture, but their values are affected by the negative lobes of the C1 highest peak and by the C2 broadband background absorption, respectively. Thus, these two peaks cannot be easily employed to evaluate the reciprocal influence of the two gases. The effects of C3 on C1 QEPAS signal can be investigated analyzing the acquired spectra, shown in Fig. 8**b**. Propane QEPAS signal (green curve in Fig. 8**b**) is negligible with respect to methane signal (red curve in Fig. 8**b**) in the whole laser tuning range. Thus, the higher value of P1 in the C1 9%:C3 0.2%:N_2_ mixture (157.11 mV) with respect to the same peak in the C1-N_2_ mixture is only due to a more efficient radiation-to-sound conversion provided by C3 presence in the matrix.

**Fig. 9:**
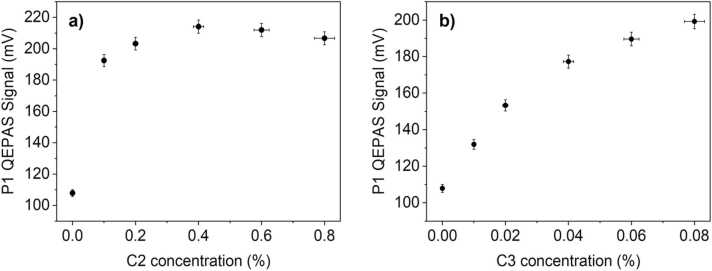
P1 peak values’ behavior for a fixed concentration of methane (8% of C1) and variable concentrations of C2 (a) and C3 (b). The error bars are calculated starting from a measured relative signal error of 2% and a relative concentration error of 4%.

The effect of C2 and C3 on C1 QEPAS signal was evaluated plotting P1 peak values against C2, C3 concentrations for several mixtures containing a fixed concentration of methane and a variable concentration of ethane and propane, respectively. The peak signals of P1 at different C2, C3 concentrations for a fixed 8% of C1 in two-component mixtures are shown in Fig. 9.

Ethane concentration was varied in the 0.1–0.8% range in the mixtures (Fig. 9**a**). As it can be seen from the graph, P1 peak value rapidly increases from 109.82 mV to 192.49 mV just by adding 0.1% of C2 in the mixture. At higher ethane concentration, P1 signal stabilizes around 200 mV. Propane concentration was varied in the 0.01–0.08% concentration range (Fig. 9**b**). The result is that P1 signal increases with C3 concentration up to 199.20 mV. Considering the C2, C3 influence on C1 QEPAS signal, a robust detection strategy must be employed to measure methane concentration in mixtures containing also ethane and propane.

### 5.1 Multivariate analysis using partial least squares regression (PLSR)

Multivariate analysis (MVA) represents a reliable solution to retrieve the concentrations of target analytes inside gas samples. Performing a statistical analysis, the selected MVA tool should be able to model and compensate the matrix relaxation effects within the different mixtures, returning the target analytes concentrations. In this work, we employed the Partial Least Squares Regression (PLSR) as MVA method. This statistical tool represents an evolution of the multilinear regression (MLR) based on ordinary least squares, sharing the basic regression model [41], [42]:

(2) Y=X∙B+E

where **Y** is the matrix of responses, i.e., the dependent variables to be calculated; **X** is the matrix of the predictors, used to retrieve the responses; **B** is the matrix of the regression coefficients; and **E** is the residuals matrix. PLSR is particularly suited for the analysis of dataset showing a strong collinearity among X values, which would affect the performance of traditional MLR. To do this, PLSR algorithm performs a decomposition of both **X** and **Y** matrix by projecting those matrices on a new space of orthogonal and independent variables, named Latent Variables (LVs), maximizing the covariance matrix **Cov**(**X**,**Y**) [42].

**Fig. 10:**
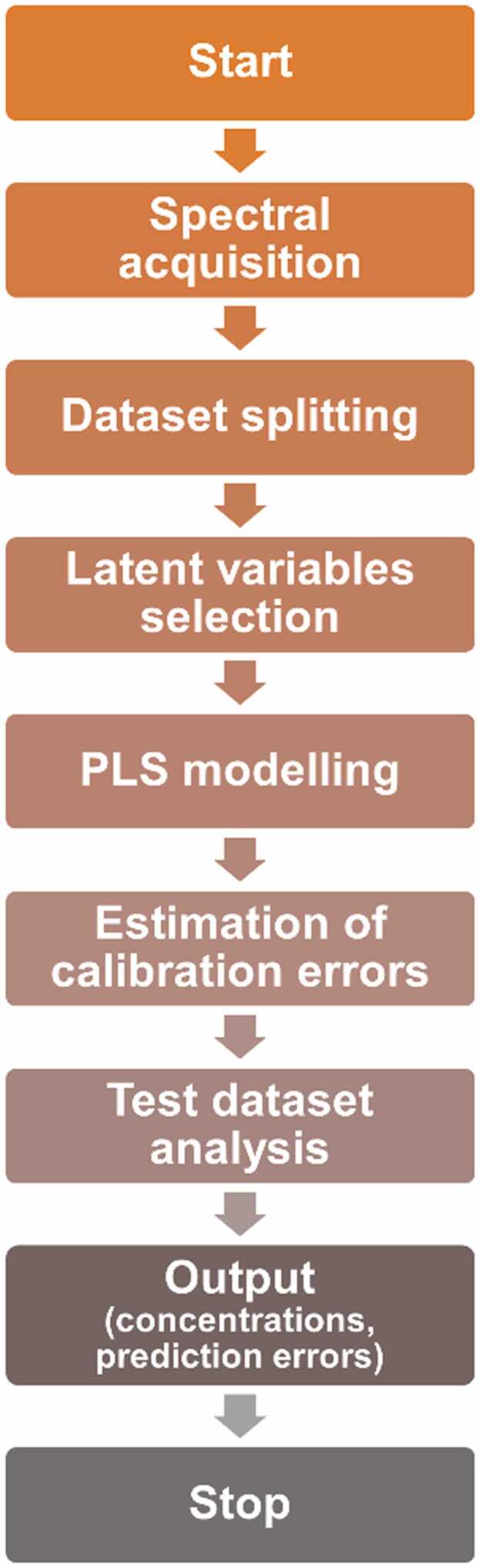
Flow chart of the developed PLSR algorithm.

PLSR has established itself as a solid method to analyze samples whose components show mutual interaction, being able to operate in different areas of chemometrics and spectroscopy. The ability of this regression algorithm to extract the truly independent factors from the analyzed datasets, makes it suitable for the retrieval of target analytes concentrations from the QEPAS spectra. The flow chart of the PLSR model developed for the analysis of natural gas-like samples is reported in Fig. 10. A MATLAB script was employed for both model construction and validation. The analysis started from the mixtures generated using the gas blender.

As first step, the collected spectra were acquired by the algorithm. Then, since PLSR can be used in a machine-learning-like approach, the collected measurements were divided in two datasets, one for calibration and one for test.

The regression model is calibrated on the training dataset using Eq. 1, and then, it is applied by means of a matrix product to a test dataset, whose concentrations are unknown to the algorithm. The calibration set was built to allow the algorithm a complete modelling of the mutual interaction among the analytes, covering the composition and the concentration range of natural gas-like samples. A total of 42 QEPAS spectra of different gas samples were collected to retrieve the concentrations of C1 and C2 in gas matrices. The measurements included single-gas spectra of C1, C2 and C3, dual-gas mixtures spectra of C1-C2 and C1-C3 and three-gas mixtures spectra of C1-C2-C3. The heterogenous composition of the acquired gas mixtures was selected to allow the regression algorithm to model the matrix effects generated by analytes with relevant and non-relevant absorption features. Indeed, even though C3 QEPAS signal is negligible with respect to other components in the mixture (Fig. 7), propane still influences the photoacoustic relaxation process of methane. Thus, its concentration must be included in the model. Conversely, PLSR filters out the signal contribution of chemical species appearing as a fixed background, such as the H_2_O used to humidify the hydrocarbons mixtures. Since both the influence on the photoacoustic relaxation and the H_2_O spectral features showed in Fig. 7 are fixed and present in all the measurements, this analyte is not recognized as a LV and thus is treated as a background offset. Three independent test sets were assembled, each one composed by 4 spectra of C1-C2 and C1-C2-C3 gas mixtures whose concentrations range of C1 and C2 matched those expected in the diluted natural gas samples (C1 > 7%; C2: 0.1–0.8%). To each selected test set corresponded a calibration set composed by the remaining 38 QEPAS spectra. As further investigation, the QEPAS spectra of a natural gas mixture with certified concentrations was acquired and blind-tested. In addition to C1 and C2, this mixture provided other molecules such as C3, C4 and CO_2_. The nominal composition of the mixtures employed as test sets is shown in Table 1.

**Table 1:** Nominal composition of the test set mixtures employed in this work.

	Mixture composition (%)					
	C1	C2	C3	C4	CO_2_	N_2_
Test set #1	**9.6**	**0.30**	**0.060**	–	–	**90.04**
	**9.3**	**0.70**	**0.020**	–	–	**89.98**
	**8.0**	**0.40**	**0**	–	–	**91.60**
	**7.0**	**0.60**	**0**	–	–	**92.40**
Test set #2	**9.2**	**0.80**	**0.010**	–	–	**89.99**
	**9.5**	**0.50**	**0.040**	–	–	**89.96**
	**9.0**	**0.80**	**0.0**	–	–	**90.20**
	**7.0**	**0.20**	**0**	–	–	**92.80**
Test set #3	**9.4**	**0.60**	**0.030**	–	–	**89.97**
	**9.8**	**0.10**	**0.080**	–	–	**90.02**
	**8.0**	**0.60**	**0**	–	–	**91.40**
	**9.0**	**0.40**	**0**	–	–	**90.60**
Certified mixture	**8.5**	**0.50**	**0.30**	**0.20**	**0.20**	**90.40**

**Fig. 11:**
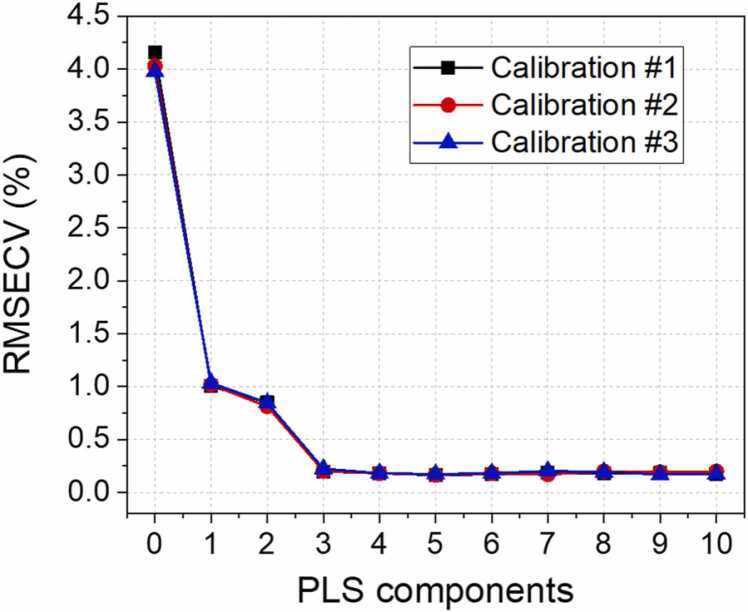
RMSECV as a function of the number of PLS components for the different calibration set employed in the analysis.

After the dataset acquisition and splitting, the number of optimal PLS components representative of the LVs was identified. This represents a crucial step, since a wrong number of components may lead to a limited interpretation of the regression model or, on the other hand, to the inclusion of non-representative factors. Many approaches can be used to determine this parameter. To validate the prediction capability of the models, the 10-fold cross-validation method was used to avoid sub- or over-fitting [43]. The PLSR calibration step was performed for all the three calibration datasets and the root-mean-square errors of cross-validation (RMSECV) obtained for each PLS components are reported in Fig. 11.

**Fig. 12:**
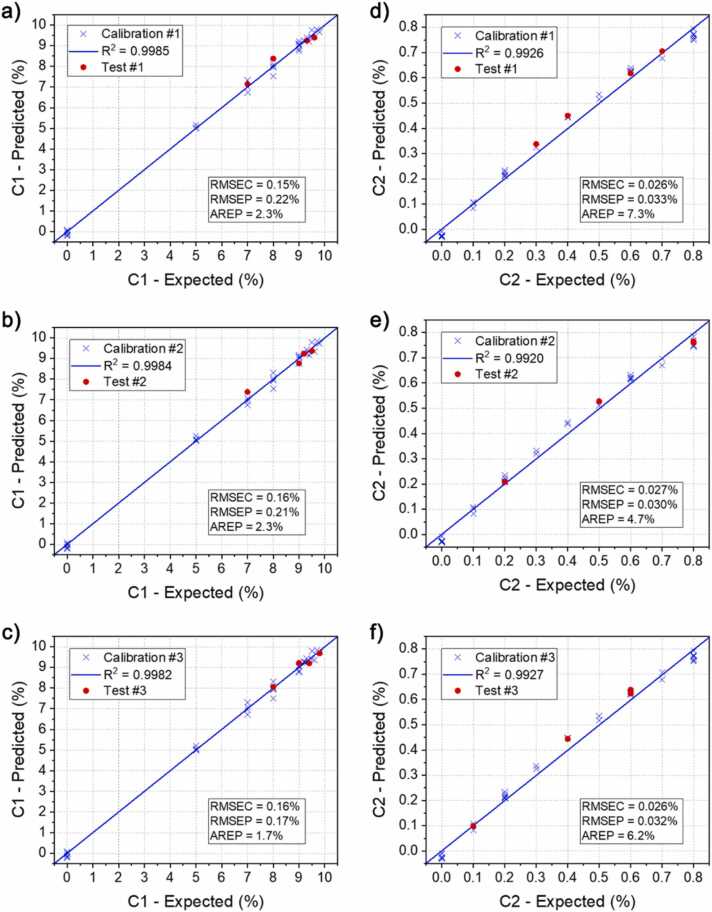
Result of the PLSR for C1, panel a-c, and C2, panel d-f. Predicted concentrations are plotted against expected concentrations for both calibration (blue crosses) and test (red dots) sets. A linear fit is superimposed to the concentrations obtained in the calibration step (blue lines) and the calculated R^2^ values are shown in the caption. The estimated RMSEC, RMSEP and AREP are reported in the graphs. (For interpretation of the references to colour in this figure legend, the reader is referred to the web version of this article.)

The optimal number of PLS components to be selected is the smallest one which provides a substantial reduction of the RMSECV. This choice prevents the occurring of overfitting in the model and provides a complete description of the collected data [43]. As it is possible to observe from the graph, the optimal number of components describing the calibration sets was found equal to 3. The percentage variance of the Y matrix explained by the model when using 3 components was > 99% for each calibration set, confirming the robustness of this choice. The identified LVs can be interpreted as the independent contribution to the QEPAS spectra of C1 and C2 plus a third component related to both the relaxation process among the analytes and the variations of the fluid dynamics properties within the sample influencing the spectrophone response. As previously mentioned, the contribution of C3 is included in the matrix effects due to the low QEPAS signal. The PLS algorithm was calibrated, and the regression coefficients matrix was calculated employing 3 components. Then, the concentrations of C1 and C2 in the test set were predicted. The obtained values were compared to the expected ones and the results of both calibration and test step are shown in Fig. 12. The errors on the concentrations retrieved in calibration and the test step are calculated as root-mean-square error of calibration (RMSEC) and root-mean-square error of prediction (RMSEP), respectively. These two parameters represent precision and accuracy of the regression algorithm [44], [45]. In addition, the average relative error of prediction (AREP) is calculated for each dataset and for each analyte. The predicted and the expected concentrations are reported in Table 2. Expected values are reported in the table alongside their uncertainty, calculated from gas cylinders uncertainty. Predicted values are reported in the table alongside the calculated RMSEC.

**Table 2:** Comparison between the predicted and the expected concentrations for the different test set and for each target analyte. Expected concentrations are reported with the calculated uncertainty, while predicted concentrations are reported with calculated RMSEC.

	C1		C2	
	Expected (%)	Predicted (%)	Expected (%)	Predicted (%)
Test set #1	9.6 ± 0.4	9.4 ± 0.2	0.30 ± 0.01	0.33 ± 0.03
	9.3 ± 0.4	9.3 ± 0.2	0.70 ± 0.03	0.71 ± 0.03
	8.0 ± 0.3	8.3 ± 0.2	0.40 ± 0.02	0.45 ± 0.03
	7.0 ± 0.3	7.1 ± 0.2	0.60 ± 0.03	0.62 ± 0.03
Test set #2	9.2 ± 0.4	9.2 ± 0.2	0.80 ± 0.03	0.77 ± 0.03
	9.5 ± 0.4	9.3 ± 0.2	0.50 ± 0.02	0.52 ± 0.03
	9.0 ± 0.4	8.8 ± 0.2	0.80 ± 0.03	0.77 ± 0.03
	7.0 ± 0.3	7.3 ± 0.2	0.20 ± 0.01	0.21 ± 0.03
Test set #3	9.4 ± 0.4	9.2 ± 0.2	0.60 ± 0.03	0.63 ± 0.03
	9.8 ± 0.4	9.6 ± 0.2	0.10 ± 0.01	0.10 ± 0.03
	8.0 ± 0.3	8.1 ± 0.2	0.60 ± 0.03	0.64 ± 0.03
	9.0 ± 0.4	9.2 ± 0.2	0.40 ± 0.02	0.44 ± 0.03
Certified mixture	8.5 ± 0.3	8.5 ± 0.2	0.50 ± 0.02	0.40 ± 0.03

First, the collected results confirm the reliability of the employed methodology. For each analyte, the RMSECs do not change, regardless of the measurements employed as test set. An average RMSEC of 0.16% and 0.026% can be calculated for C1 and C2, respectively. The RMSEPs show a slight discrepancy among the employed tests, corroborating the assumption of an unbiased selection of the test measurements. An average RMSEP of 0.20% and 0.032% can be calculated for C1 and C2, respectively. The calculated AREP are < 2.5% and ~6% for C1 and C2, respectively. The retrieved C1 concentrations fall within the confidence interval provided by the uncertainties of the expected mixtures, while the retrieved C2 concentrations are slightly outside. The larger discrepancy of C2 compared to C1 can be ascribed to the nonlinear response of the QEPAS sensor due to the Lambert-Beer law for non-weak absorptions. It is worth to underline that the PLSR algorithm is able to properly operate on models that are linear, or that does not appreciably deviate from the exact linearity. When this condition is not met, and significant nonlinearities occur, the algorithm may lose its predictive power. However, even though the nonlinear response is clearly visible in the calibration curve of peak P1 of C1, this leads to a saturation of the QEPAS peak signals in the concentration range employed for the analysis (C1 >7%), as shown in Fig. 4**a**. Therefore, the algorithm ignores the information provided by the nonlinear peaks, and rather focuses on the non-saturated features, showing a linear response (Fig. 4**b**). On the other hand, the C2 calibration curve show a moderate nonlinearity for the most intense absorption peak (Fig. 6**a**), without signal saturation. Therefore, the algorithm still considers these information and is not able to correct the observed nonlinear trends. In fact, the results of PLSR calibration presented in Fig. 12**d-e-f** show a slight nonlinearity analogous to the one showed in Fig. 6**a**, and the retrieved test concentrations align with the calibration dataset. This determines a lower accuracy for C2 concentration measurement compared to C1 but does not compromise our investigation, as expected from literature [46]. The RMSEC and RMSEP values obtained for C1 are compatible with the measured relative fluctuation of 2% in the QEPAS signal, highlighting the efficiency of the employed regression algorithm. Conversely, the values obtained for C2 are higher compared to the QEPAS signal fluctuation. As previously discussed, this discrepancy can be ascribed to the nonlinearities determined by power losses due to direct absorption.

In Table 2, the results of the analysis operated on the certified natural gas mixture, diluted 1:10 in N_2_, are also reported. In this case, the employed calibration dataset consisted of all the 42 QEPAS spectra collected and the optimal number of PLS components was equal to 3. The obtained calibration errors are analogous to those previously estimated. As results of this blind test, the retrieved C1 concentration showed an excellent agreement with the one expected, while the retrieved C2 concentration showed a larger discrepancy compared to C1. The QEPAS signals of the C2 absorption peaks in the natural gas sample correspond to those collected in calibration step for the mixture containing only 0.4% of C2 in N_2_. Therefore, this discrepancy can be ascribed to the actual C2 concentration inside the gas cylinder employed for calibration, since C2 did not pointed out any relevant matrix effect related to heavier hydrocarbons. Moreover, the arisen of unpredicted matrix effect should have been recognized in QEPAS signal of C1, being the lightest hydrocarbon and the most influenced by the matrix composition, while no significant discrepancy was observed.

## 6 Conclusions

In this work, the challenge of photoacoustic detection over a wide concentration range was taken up for the analysis of natural gas-like mixtures. Natural gas, with all the alkanes as well as non-hydrocarbon components, represents a perfect example of a complex and widely variable gas matrix. In particular, the detection and quantification of methane ethane and propane, provide useful geochemical fingerprints for characterization of oil and gas reservoirs. In this context, the intrinsic problem for photoacoustic generation, concerning the signal dependence of a target molecule on the relaxation dynamics within the gas matrix, has been deeply investigated for C1, C2, C3 concentrations exceeding the part-per-thousand concentration range up to several percent. QEPAS technique was the photoacoustic approach exploited. For safety reasons and to prevent the contamination of the sensor, the hydrocarbon mixtures generated for this analysis were diluted 1:10 with pure nitrogen. The architecture of the sensing system has been largely improved and the data acquisition automatized, thanks to a Red Pitaya STEMlab 125–14 board to both control the laser source and demodulate the collected QEPAS signal. The whole system consists in a shoe-box sized QEPAS sensor, ready to deploy for in situ operations. The study of the photoacoustic generation at high concentrations focused on C1, C2 signal behavior with variable C1, C2, C3 mixtures. In particular, C1 and C2 QEPAS calibrations showed both linear and nonlinear trend, and the presence of C2 and C3 in mixtures strongly affected the C1 signal by influencing its relaxation dynamics. In order to address this issue, we demonstrated that PLSR, employed as a statistical method, is capable of accurately extracting methane and ethane concentrations and filtering out the influence of the matrix variation, in terms of photoacoustic relaxation effects and variation in the properties of the fluid affecting resonance frequency and Q-factor of the resonator. The reliability of this method was further validated calculating C1, C2 concentrations over a large set of C1, C2, C3 mixtures, simulating natural gas samples diluted in nitrogen. The performances reported in this work allowed us to demonstrate the potentiality for the QEPAS technique to detect methane and ethane over a seven decades dynamic range, from ppb scale up to percent scale, without experiencing a sensible accuracy and precision degradation due to fluctuating backgrounds. With the aim of developing a reliable sensor for the analysis of natural gas composition and isotopologue ratios, further investigations on the effects of temperature variations on the sensor response will be performed in future works.

## Declaration of Competing Interest

The authors declare that they have no known competing financial interests or personal relationships that could have appeared to influence the work reported in this paper.
